# Diabetes Mellitus: Indigenous naming, indigenous diagnosis and self-management in an African setting: the example from Cameroon

**DOI:** 10.1186/1472-6823-9-5

**Published:** 2009-02-19

**Authors:** Paschal K Awah, Nigel C Unwin, Peter R Phillimore

**Affiliations:** 1 HoPiT Research Group, PO Box 8046, Yaounde, Cameroon; 2 Department of Anthropology, Faculty of Arts, Letters and Social Sciences, University of Yaounde I, Yaounde, Cameroon; 3 Centre for Population Studies and Health Promotion, PO Box 7535, Yaounde, Cameroon; 4 Institute of Health and Society, Faculty of Medical Sciences, Newcastle University, Newcastle, NE2 4HH, UK; 5 School of Geography, Politics and Sociology, Faculty of Law and Social Sciences, Newcastle University, Newcastle, NE1 7RU, UK

## Abstract

**Background:**

The objective was to examine how the indigenous naming, indigenous self-diagnosis and management of diabetes evolved with awareness in order to develop a socially oriented theoretical model for its care.

**Methods:**

The data was collected through a one-year extended participant observation in Bafut, a rural health district of Cameroon. The sample consisted of 72 participants in a rural health district of Cameroon (men and women) with type 2 diabetes. We used participant observation to collect data through focus group discussions, in depth interviews and fieldwork conversations. The method of analysis entailed a thick description, thematic analysis entailing constant comparison within and across FGD and across individual participants and content analysis.

**Results:**

The core concepts identified were the evolution of names for diabetes and the indigenous diagnostic and self-management procedures. Participants fell into one of two naming typologies: (a) Naming excluding any signs and symptoms of diabetes; (b) naming including signs and symptoms of diabetes. Participants fell into two typologies of diagnostic procedures: (a) those that use indigenous diagnostic procedures for monitoring and controlling diabetes outcomes and b) those that had initially used it only for diagnosis and continued to use them for self management. These typologies varied according to how participants' awareness evolved and the impact on self-diagnosis and management.

**Conclusion:**

The evolution of names for diabetes was an important factor that influenced the subsequent self-diagnosis and management of diabetes in both traditional and modern biomedical settings.

## Background

According to the World Health Organisation, the term diabetes mellitus describes a metabolic disorder of multiple aetiology characterised by chronic hyperglycaemia with disturbances of carbohydrates, fat, protein metabolism resulting from defects in insulin secretion, insulin action or both [1]. In many developing countries, where diagnostic facilities may be minimal, primary healthcare facilities lack the basic diagnostic facilities to screen and classify people living with diabetes or at risk of developing diabetes. For this reason, people turn to traditional diagnostic methods linked to the names that are used in calling the disease. These classifications are based on their definition of the presence of physical, psychological and social signs and symptoms prevalent at the time of diagnosis [2].

Diabetes Mellitus, particularly Type 2 diabetes is a growing health problem in Africa. Diabetes has an age-adjusted prevalence rate of between 1–10% in rural and urban areas [3-5]. The World Health Organization estimates that in 2000 there were 7.1 million people with diabetes in sub-Sahara Africa, and that by the year 2030 the number will have increased to 18.6 million. The overall prevalence of diabetes in traditional rural African communities is less than 1%, but rises to as high as 20% in some adult subgroups above 20 years in some African cities [1,5]. In Cameroon and Tanzania, the rate is about 5% [6,7].

One of the main problems that impede the management of diseases in Africa is the acknowledgement of that disease and the recognition of the risk factors that underlie it. The treatment for diabetes faces covert and overt resistance by any means, and also a reluctance of many people to be convinced by the public health naming and classifications. This severely affects the perceived effectiveness of biomedical approaches used to provide diabetes care. This paper is intended to explore some of the major indigenous naming, diagnosing and self management of diabetes and their implications to public health care in contemporary Africa. We will focus on the evolution of names of diabetes as people's awareness progress. Using phrases and taxonomies, we will explore the naming, diagnosis and self management of diabetes from an indigenous perspective.

## Methods

### Setting

Bafut is a rural health district situated about 285 miles to the Northwest of Yaounde, the capital city of Cameroon. Bafut has a total land area of about 2847 square miles with an average density of about 14 inhabitants per square kilometre. According to the health census of 1999, it has a population of 74,750 inhabitants. Bafut is a typical area where state structures at the local level stand alongside, or overlap with traditional structures. The traditional structures have existed since the founding of this local kingdom. Though three main languages (Bafut, English and Pidgin English) are used for daily communication, Bafut is the most frequent. There are ten health units distributed in eight health areas. Each of the health units has a pharmacy run by the North West Special Fund for Health that should ensure the availability of diagnostic procedures and drugs in all health units of the public and private sectors. In 2003 there were four medical doctors and 20 nurses in the health district.

### Design

The design of the study was qualitative, using participant observation as the field data collection method, within which focus group discussions, in-depth interviews and conversations formed part of the array of methods. The study lasted for over two years, from June 2001 to June 2003.

### Sampling

The sampling of fieldwork participants was both purposive and convenient. A purposive sample selection was used to select participants knowledgeable in the understanding of the Bafut Language to participate in focus groups and in-depth interviews and also in their ability to link the names used in addressing diabetes to diagnosis and management of diabetes. The convenient approach was used in conversations where chance events introduced interaction with people.

### Participants

Seventy-two diabetes patients were involved in this study. A total of six focus group discussions and fourteen in depth interviews were conducted, including ten selected fieldwork conversations from the database of conversations.

#### Data collection methods and procedures

Participant observation was the method used for data collection. Some of the methods used and whose data is reported in this paper involved fieldwork conversations, focus group discussions and in depth interviews.

#### Conversations

Numerous fieldwork conversations [8-12], were held with participants using an informal style of questioning. These were event and case driven, to make an easygoing dialogue possible. However, simply joining-in conversations that people were holding was the easiest way to go about generating them and respecting participant's points of view [11]. This led to confidence building and identification of themes and concepts. It became easier to probe into certain topics where gaps in knowledge existed and of conflicting viewpoints necessitating more understanding.

#### Focus group discussions (FGD)

Focus groups are a form of group discussions that capitalise on communication between research participants in order to generate data [13]. Although group discussions are often used simply as a quick and convenient way to collect data from several people simultaneously, focus groups in this study explicitly used group interaction as part of the method of participant observation. People were encouraged to talk to one another: asking questions, exchanging anecdotes and commenting on each others' experiences and points of view. The method was useful for exploring people's knowledge and experiences, and was used to examine not only what people think, but also how they think and why they think in a particular way. The focus groups linked to the overall process of continuous data collection, as inroads were made into people's daily lives verifying issues of naming and diagnosis that were raised in focus groups dynamics.

The participants in focus groups were purposively selected and the number of participants in focus groups varied from six to eight. We sought to make the sessions relaxed: in a comfortable setting, with refreshments, and sitting round in a circle to establish an enabling environment. FGD were organised in public conference rooms. Sessions lasted from one and a half to two hours but always ended up in social drinking where some of the opinions left out by the more timid participants in the groups gradually emerged. An interventionist style was used to urge discussions to continue beyond the initial questions, and encouraging the group to discuss the inconsistencies both between themselves and within their own thinking. We used disagreements within groups to encourage participants to elucidate their points of views and to clarify why they thought as they did.

We used focus groups because they provided a way to help people explore and clarify their views in ways that might be less easily accessible in a one-to-one interview and in conversations. Focus groups reach the parts that other more formal methods may not reach; revealing dimensions of shared or tacit understanding that often remain untapped by the more formal social science research methods and fitting in well therefore to the ethos and goals of an ethnographic approach [14]. On the other hand, the shortcoming of such group dynamics is that the articulation of group norms may well silence individual voices of dissent.

#### In-depth interviews

A series of open-ended questions were used to guide and encourage individuals to explore the issues of importance to them, in their own vocabulary, generating their own questions and pursuing their own priorities. The one-to-one interactions enabled participants to work alongside the investigator, generating and taking the themes in new and often unexpected directions. They helped the probing into the different ways of communication that people used in day to day interaction, including jokes, anecdotes, teasing, and arguing. Gaining access to such variety of communication was useful because people's knowledge and attitudes are not entirely encapsulated in reasoned responses to direct questions. Tapping into such interpersonal communication was also important because this highlighted cultural values and group norms that shaped the evolution of fieldwork.

#### Data management and analysis

All data were tape recorded, transcribed and compared with notes taken during field data collection. Transcribing permitted the identification of new emerging concepts providing new and follow-up questions for subsequent fieldwork. Data was transcribed and translated. All local remarks presented in this paper, as direct quotations are verbatim extracts from these transcripts. There were about 3000 pages of text from transcripts of observation notes, conversations, interviews and focus group discussions relating to the topic of this paper.

A thick description of text has been used to analytically understand and interpret this data. These analyses facilitate a thick description, determining the main themes that emerge at all stages of fieldwork, and obtaining the meanings that are attached to them. The 'thick description' has necessitated the sorting of the meanings inherent in data. By so doing, the symbolic values attached to every naming, diagnosis and self-monitoring have been reconstructed and represented with respect to the participants' lay interpretation, construction and understanding. Through analysing the operation of humour, consensus and dissent, and by examining different types of narratives used within the group and through the other methods used and shared, common knowledge has been identified [14,15].

#### Ethics and consent

An ethical clearance was obtained from the Cameroon National Ethical Committee and an authorisation to conduct research from the Ministry of Scientific Research of Cameroon for fieldwork. The district medical officers were informed and local administrative authorisation obtained for the project before fieldwork started. Informed consent and voluntary participation were sought from people with diabetes to observe them in their daily lives. The informed consent process was continuous until the end of fieldwork. Pseudonyms are used to protect the identities and privacy of participants, taking liberty with ethnographic facts, in order to disguise identities, doing all to retain contexts and arguments.

## Results

### Socio-demographic characteristics

Table 1 summarises the socio-demographic of participants and the composition of participants by methods.

**Table 1:** Distribution of study participants by some socio-demographic of participants and the composition of participants by methods.

	Research methods		
	
**Characteristics of patients**	Focus Group Discussions n = 6	In Depth Interviews n = 14	Fieldwork conversations n = 21
	No of participants = 37	No of participants	Number of participants = 21
Median age/Age ranges	52 (40–75)	57 (48–75)	56 (42–75)
			
FGD by Gender			
Male	12	6	12
Female	12	8	15
Mixed gender	13	NA	NA
			
Marital status			
Married	33	13	8
Widow	4	2	2
			
Occupation			
Civil servant	5	2	2
Farmers/Other artisans	15	4	4
Housewives	8	4	2
Retired	4	4	2

### Naming of diabetes

In every culture a number of words are always used to identify and name an illness, so it happens with diabetes. In many cases, there are no straight-cut words to do so but a series of phrases describing the signs and symptoms that suspect or diagnose illness. In a fieldwork conversation with people living with diabetes, some patients attempted all the possible ways of naming diabetes as the following quote amplifies.

Ambi. 'We call diabetes *Nighoni-shugar*.'

Samoa. 'It means sugar, sugar sick.'

Pet. 'With the word sugar, in Bafut, this is just a new thing that we have inherited from the medical department. We call it *Kweeh, Kukwée *to mean an illness that originates from sugar and too much sweet things.'

Pastir. 'From the traditional point of view we did not have diabetes. It has been unknown to us. It might have been there but unknown to us. We call it *Nighoni-shugar*'

Samoa. '*Fumbgwuang, Nshugar *is how we commonly call it. It is *Nighoni-Fumngwuang*. Otherwise, we call it *Nighoni-shugar*.'

Pete. '*Nshugar à sesang*.'

Ambi. '*A sefune Leleh*.'

Pettia. *Fumbgwuang ghanée*.

(Extract from a conversation with diabetes patients).

The above quote presents the naming and taxonomy of diabetes. The original language of the quote was Bafut. But the words describing diabetes are maintained. None of the names in the above quote can be taken as a straightforward equivalent for diabetes. They are descriptions of participants' understanding and naming of diabetes. Out of a number of terms in use in Bafut, two key words, *fumbgwuang *and *shugar *are most frequently heard. These are used along with *Nighoni*. *Nighoni-shugar *denotes 'sugar disease' and *nighoni-fumbgwuang *'disease that is sweet'. Thus one refers to a food product, the other to taste, with *nighoni *meaning sickness or illness. Yet, while *shugar *is sugar, *fumbgwuang *means salt, as well as referring to a sweet taste, a taste that goes beyond the sweetness associated with sugar. Thus salt and sugar are associated with diabetes. But perhaps most relevantly, the disease is seen as linked to an indigenous sense of sweet taste, which encompasses the more familiar salt. In this manner, diabetes is the illness that comes from sweetness and the illness coming from sugar. However diabetes is widely and often used especially when referring to it in Western languages like English and French or in a lingua franca – Pidgin English.

But it is important to emphasise that these ideas and responses are not static or unchanging. Indeed, as more and more patients are clinically diagnosed, the word diabetes becomes increasingly part of common currency in localities, and may well in due course completely supplant the local terms discussed above. Nevertheless, when people were asked to explain what they understood diabetes to be, it was the local terms and their connotations that were most readily called upon. In this way, diabetes is still most commonly understood and made sense of by reference to traditional or pre-existing ideas. The use of the word diabetes cannot be assumed to mean that the user thinks of diabetes as healthcare providers might hope. Nonetheless, over time, that may be expected to happen. While we have discussed the connotations of the two popular idioms used in describing diabetes in Bafut, there are other indigenous terms and descriptions that are nowadays fading from use as the following quote illustrates:

Diabetes was called *yoongkee *and *ki-yoongkee*. But we do not more call it like that today. Only very old people like myself call that name. Even when they refer to diabetes as *Nighoni-Shughar *it does not have the same complete meaning to what they are referring to.

(Male patient of diabetes)

**Figure 1:**
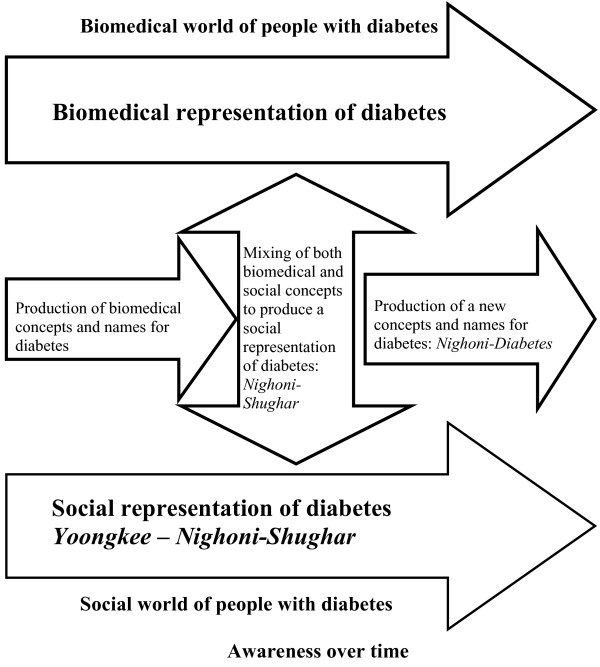
**Schematic reconstruction of the taxonomy for diabetes**.

*Yoongkee *or *Ki-yoongkee *is an illness description in Bafut where one's blood is believed to transform into water and one develops swollen feet and a swollen stomach. It may be related to foot complications of diabetes, but can also relate to other health issues associated with lower limbs oedema and ascitis. It is a clear evolution from words and phrases not containing signs and symptoms of diabetes, to idioms containing signs and symptoms and perceived causes. We would not be surprised if, one day, *shugar and fumbngwuang *are replaced by diabetes, to be called *nighoni-diabetes*. Figure 1 illustrates the evolution of taxonomy for diabetes as awareness increases.

Terminology, and the understanding of that terminology about diabetes, is thus constantly evolving not only in Bafut but in most African languages as was illustrated during the phase of the urban ethnographic fieldwork of this study. How much is changing? Though people remain faithful to traditional cultural values about the causes and treatment of diabetes, several changes are apparent. Firstly, the naming of diabetes has moved from purely traditional appellations to a mixture of local languages and English or French whereby high glucose in the blood is associated with diabetes. This calls for the use of local language words as prefixes, '*Nighoni-Shugar*' by Bafut people. There is every indication that the more signs and symptoms of diabetes are acknowledged and accepted by people, the more the names will evolve and the local prefix may eventually fade and be forgotten.

### Diagnosis and monitoring of diabetes

#### Self-diagnosis

Irrespective of the existence of modern healthcare facilities in both urban and rural African settings, many people still use traditional/indigenous self-diagnostic tools as illustrated in the quotes below:

Before I could know that I have diabetes, I tasted my urine when I was sick. When the urine tasted sweet I began to suspect that I had diabetes. That now helps me to control the level of sugar in my body.

Extract from a conversation with a male diabetes patient

I was always feeling thirsty and drinking water, then a friend told me that I should be having diabetes. When I turned up at the hospital to consult, I complained about the thirst. The doctor sent me to the lab for a urine and blood test. Finally, he informed me that I had diabetes. Today, when my blood glucose is high I am able to know because I start feeling thirsty and drink a lot of water.

Extract from a FGD with female diabetes patients.

The above quotes illustrate some of the main indigenous methods of diagnosing and controlling diabetes. If a person suspects that he may be ill from diabetes, a common way is by self-diagnosis. One of the most common ways is by tasting his/her own urine. When one urinates frequently and suspects that something is wrong, so one samples one's urine. If it has the taste of sugar, or tastes sweet, then a person suspects diabetes.

I used to urinate frequently, especially in the night. I would go out and urinate and after about an hour or two my bladder will be full again and I will continue like that till dawn. Some people told me that I was washing away dirt in my stomach. One day, I was travelling and in the course of a conversation, my neighbour in the car told me that it was not normal and that I may be sick with diabetes. I did not know what that was. When I returned to, I consulted at the hospital and was diagnosed with diabetes.

Extract from an IDI with a female diabetes patient.

Indeed, individuals who are already being treated for diabetes sometimes use these methods to help diagnose others and also to control their blood glucose levels, especially when they lack the means to do it in a clinic.

Urinating also provides another clue to self-diagnosis as the following quotes illustrate:

Paat: I urinate on the ground where I can easily see the trace of urine even after it dries off. If after some time I witness ants feeding on my urine, I suspect that my blood glucose is high. It is the same way that I came to be aware that I had diabetes after consulting with a traditional healer.

Extract from an IDI with a male diabetes patient.

In Cameroon, people urinate wherever it is convenient. If, when one urinates, one then finds that ants are visiting the spot to feed on the urine, one is likely to conclude that one suffers from diabetes. People relate that observation to their observation of ants when they crowd around deposited particles of salt or sugar. Also, the foaming of urine is diagnosed as possibly being an indicator of diabetes or uncontrolled diabetes.

...When your urine foams a lot, it is a sign that you are not well and that you may be having diabetes.

Quote from a conversation with female diabetes patient.

### Diagnosis by divination

Besides the practical aspects, divination is another indigenous approach used to diagnose diabetes as illustrated by the following quote:

...When Ambrose, aged 42, was diagnosed with diabetes, his family consulted a diviner, who concluded that the cause could be traced back to an ancestor...

Extract from a fieldnote.

The revelation of the diviner in the illustration above helped to affirm the diabetes status. Revelations from diviners are not only limited to ancestors but also to witchcraft. Ancestral and witchcraft invocations and declarations are treated simultaneously as emergencies and truth. Traditional healers are often brought in to perform their own 'diagnosis' before or after a person has first been given the diagnosis of diabetes at a hospital and mainly at the request of traditional healers. Firstly, episodes of unexplained untreated illnesses may prompt a person to seek explanation from a traditional healer. When that occurs, the traditional healer may use the above-mentioned diagnostic procedures to seek to determine the illness, but will colour it with divination. His divination may diagnose a living agency – witchcraft – or a supernatural one – ancestor – as those responsible for its occurrence. In some cases, traditional healers' knowledge of some of the signs and symptoms help them to diagnose that a person is diabetic. When the healer divines, s/he often encourages the person s/he is treating to attend a modern clinic for confirmation or tests. A traditional healer who refers a patient to a health unit for the confirmation of diagnosis does this only when he is very sure that his diagnosis would not fail. Secondly, diagnosis may start at the hospital but diagnosis at the hospital reveals to the patient that the signs and symptoms he or she is suffering from is diabetes. But what it fails to explain is the cause of the diabetes and why it has affected that person and not another. For that reason some people turn elsewhere to seek the 'ultimate' diagnosis from traditional healers, because it is widely believed that all illnesses have an underlying cause independent of the affected individual.

### Self-management

Diagnosis by divination helps to inform people with diabetes about their illness and reinforces their help seeking behaviour to treating themselves either through traditional medicine or through biomedicine. However, this strongly depends on the ability of the traditional healer to orientate the person to any of the treatment pathways. The take home message of every patient is that an illness known as diabetes or an illness resulting from raised sugar (sugar illness) is in the body and needs to be treated or cured. The willingness to treating diabetes takes the person with diabetes to a biomedical clinic and the willingness to curing diabetes takes the person to a traditional healer.

## Discussion and conclusion

We started from a biomedically defined syndrome (diabetes) and sought to understand how this disease was defined, located and diagnosed in the thought and help seeking behaviour of people with diabetes and the people living in Bafut. The process of doing justice to this local knowledge meant casting aside the parameters of the initial syndrome altogether. The boundaries of what is or is not diabetes, in biomedical terms, disappeared in this perspective. This has, likewise, meant moving beyond the biomedical naming of a disease. Not only does this involve a shift from a focus on a disease but also to a focus on the experience of illness. As in Robert Pool's [11] case, though probably not to the same extent, our enquiries also redefine the naming and diagnostic patterns of diabetes. It is also that these changes in taxonomies reshape the nature of the supposed entity in question. Thus there is not an indigenous naming category of illness or disease that occupies the same space as 'diabetes' in biomedicine.

The above may not be a complete representation of all the indigenous approaches in Cameroon, but are of the most popular of them. Whereas Diabetes Mellitus conveys little about the signs and symptoms of the disease to participants, the two indigenous terms are grounded in the experience of the person afflicted – the experience of illness – and also suggest crucial things about the underlying mechanisms of illness and its origin. These diagnosis and interpretations help patients to make some sense of what diabetes means for the body. Yet we also note here that urination can be ambiguous as an informal diagnostic tool. For urinating frequently is also said to be a way of washing out sickness, and therefore, a sign of getting rid of a source of ill-health, rather than as a clue to an emerging health problem.

That urine tastes sweet means that a person has the 'sugar illness'. The sampling of urine, either through its taste or by its foaming after urination or visitation by ants, were back-ups for fasting blood glucose (FBG) test in the absence of money to pay for the test. When patients do this, it enables them to measure qualitatively the magnitude of FBG without resort to figures or clinics. They can stay home until they have enough money to attend the clinics. In other instances, some patients turn up at the clinics when signs and symptoms of discomfort resulting from diabetes are 'confirmed' in urine. We see here the ability of some patients to self-monitor blood glucose by this method.

It is worthwhile stressing how much the linked events of death, succession and conflicts explained through the divination mark a transition that is crucial to an understanding of beliefs about ancestral and witchcraft influences in the appearance (or 'reappearance') of diabetes. But what makes a traditional healer confidently explain after divination that a patient is diabetic? When a patient explains symptoms, without being aware that these are indicators of diabetes, many healers can straightaway diagnose diabetes – but this is the crucial difference from the clinic, not as a straightforward illness, but instead as the outcome of a conflict. Performing a diagnostic ritual is not limited to revealing diabetes, but also the agency responsible for causing diabetes.

Self diagnosis and diagnosis by divination are good indicators for people with diabetes to understand that the signs and symptoms they are having are signs and symptoms of diabetes. They served as the basic relay to informing and steering them to seek help in treating the illness which they had identified and named as diabetes.

Because of the evolving rates of prevalence, morbidity, and mortality among Africans south of the Sahara, type 2 diabetes is a major health problem that health care providers should address with consideration for cultural values. A critical factor in health outcomes for diabetes is self-diagnosis and self-management that leads to metabolic control of blood glucose levels (United Kingdom Prospective Diabetes Study [16]. Self-management, defined as the knowledge and skills necessary to take care of oneself, manage crises, and change one's lifestyle to manage illness successfully [17,18], is an important aspect of controlling blood glucose levels. A major self-management goal set for individuals with diabetes by health care providers regarding self-management of diabetes is tight control of blood glucose levels through adherence to a protocol of blood glucose self-monitoring, diet, exercise, and medications [19,20]. However, this prescribed regimen includes a system of surveillance that makes self-management very complex. Biomedical regimens become more complex when indigenous concepts and values are added to them [21]. Thus, interventions are needed that help Africans to recognise the signs and symptoms of diabetes and for those who live with it, improve their self-management and reduce the morbidity and mortality associated with it. To be effective, interventions that facilitate self-management need to be closely linked to African's cultural beliefs, values and practices. An important cultural resource for Africans that might affect self-management of diabetes is the indigenous naming, beliefs, diagnostic and monitoring procedures. These are embedded in the rich cultural heritage of Africans [22-25] and warrant careful study and integrating into interventions.

This study shows the importance of indigenous naming and diagnosis in self-management of diabetes. Further research is required to focus on participants who have other chronic illnesses to research on whether it is the same pattern of disease naming and diagnosis that prevails. More data collection and analysis need to be done with a larger sample to develop this model fully. Little is still known, in general, about how the process of naming and diagnosis affects self-management of chronic illness in general. Further research should also focus on participants who are members of a variety of cultural backgrounds, especially using more or larger language groups. There is a need to extend this research to Africans who are not tied to a specific language or ethnic groups. Although we described in this study the process of how naming and diagnosis affect self-management of diabetes, the ultimate need is to develop and test interventions related to indigenous naming, diagnosis and monitoring of chronic illness. More descriptive work needs to be done to develop such studies. For example, this study has shown that for some Cameroonians, the naming and diagnostic practices are important clues in diabetes self-management. However, the frequency of such practices is unknown. Which of the diagnostic procedures give support in self-management? A pilot survey using questionnaires could provide answers to such questions. Furthermore, information is needed regarding whether interventions should be focused on the individual or groups and the best settings for conducting interventions.

## Conclusion

For many Africans, indigenous diagnosis like divination and others forms grounded in people's cultures is an important diagnostic tool of illness, therefore believed to provide clues that guide the naming, diagnosis and management of their diabetes. One biomedical implication is that consultants and researchers should assess these beliefs and practices of their patients and then explore how they might affect self-management of diabetes. If, on assessing, researchers and consultants find that indigenous diagnostic practices are important to patients, they can encourage these individuals to use these practices to provide strengths and motivations to take care of themselves.

## Abbreviations

FBG: fasting blood glucose; FGD: Focus Group Discussions; IDI: in-depth interviews; UKPDS: United Kingdom Prospective Diabetes Study.

## Competing interests

The authors declare that they have no competing interests.

## Authors' contributions

This study was conceived by PA for his Ph.D fieldwork and supervised by PP and NU. PA conducted fieldwork data collection. We have all made a substantial contribution to (a) the conception and design, analysis and interpretation of data; (b) drafting and revising the article; and (c) approval of the final version. We all take public responsibility for the work.

## Pre-publication history

The pre-publication history for this paper can be accessed here:


